# The genome of the Black Bengal goat (*Capra hircus*)

**DOI:** 10.1186/s13104-019-4400-3

**Published:** 2019-06-27

**Authors:** Amam Zonaed Siddiki, Abdul Baten, Masum Billah, Mohammad Atique Ul Alam, Kazi Shefaul Mulk Shawrob, Sourav Saha, Muntaha Chowdhury, Atif Hasan Rahman, Michael Stear, Gous Miah, Mahadia Kumkum, Mohammad Sirazul Islam, Mohammad Alamgir Hossain, A. K. M. Moniruzzaman Mollah, Md. Kabirul Islam Khan

**Affiliations:** 1 Genomics Research Group, Department of Pathology and Parasitology, Faculty of Veterinary Medicine, Chattogram Veterinary and Animal Sciences University (CVASU), Chattogram, 4225 Bangladesh; 2 AgResearch, Private Bag 11008, Palmerston North, 4410 New Zealand; 3 Department of Computer Science and Engineering, Bangladesh University of Engineering and Technology (BUET), Dhaka, 1000 Bangladesh; 4 Department of Animal, Plant and Soil Sciences, School of Life Sciences, AgriBio, La Trobe University, Bundoora, VIC 3083 Australia; 5 Department of Genetics and Animal Breeding, Faculty of Veterinary Medicine, Chattogram Veterinary and Animal Sciences University (CVASU), Chattogram, 4225 Bangladesh; 6 Department of Biological Sciences, Asian University for Women (AUW), Chattogram, 4000 Bangladesh; 7 Southern Cross Plant Science, Southern Cross University, Lismore, NSW 2480 Australia

**Keywords:** Black Bengal goat, *Capra hircus*, Whole genome sequencing, Assembly, Annotation

## Abstract

**Objectives:**

Black Bengal goat (*Capra hircus*), a member of the Bovidae family with the unique traits of high prolificacy, skin quality and low demand for food is the most socioeconomically significant goat breed in Bangladesh. Furthermore, the aptitude of adaptation and disease resistance capacity of it is highly notable which makes its whole genome information an area of research interest.

**Data description:**

The genomic DNA of a local (Chattogram, Bangladesh) healthy male Black Bengal goat (*Capra hircus*) was extracted and then sequenced. Sequencing was completed using the Illumina HiSeq 2500 sequencing platform and the draft assembly was generated using the “ARS1” genome as the reference. MAKER gene annotation pipeline was utilized to annotate 26,458 gene models. Genome completeness was assessed using BUSCO (Benchmarking Universal Single-Copy Orthologs) which showed 82.5% completeness of the assembled genome.

## Objective

Black Bengal goat (BBG) belongs to the Bovidae family and found throughout Bangladesh, West Bengal, Bihar, and Orissa regions of northeastern India. It is estimated that more than 90% of the goat population in Bangladesh comprised the Black Bengal, the remainder being Jamunapari and their crosses [1]. Higher prolificacy, fertility, resistance against common diseases, adaptability to the adverse environmental condition, early maturity, seasonality and superiority in the litter size are some of the outstanding features of BBG. Besides, it produces excellent quality flavored, tender and delicious meat with low intramuscular fat and fine skin of extraordinary quality for which there is tremendous demand all over the world [1, 2]. Moreover, it plays a vital role in the economy of Bangladesh by contributing 1.66% of the GDP (Gross Domestic Product) (DLS 2017).

Fortunately, the market demand of Black Bengal goat is emerging. This gives breeders of original/rare breeds an opportunity to expand the stock and preserve its genetic diversity. One of the primary goals in managing goat populations is to maintain high-level genetic diversity and low-level inbreeding. To estimate the future breeding potential of a goat breed, it is necessary to characterize the genetic structure and evaluate the level of genetic diversity within the breed. Moreover, a long term genetic approach can be used to improve the spectacular economic characteristics of BBG [3].

Therefore, the genetic characterization of the entire BBG genome is essential in characterizing its economic traits as well as adaptive capability. With the availability of whole genome sequence, the targeted areas for genetic improvements are now: goat prolificacy, growth rate, meat quality, skin quality, disease resistance, and survivability. A complete and accurate reference to the goat genome is an essential component of advanced genomic selection of product characteristics.

## Data description

At first, A 3 years old male healthy Black Bengal goat (BBG) without known genetic diseases was selected for blood collection. Genomic DNA from each animal was isolated from the EDTA-blood, using the Addprep genomic DNA extraction kit (South Korea) (detailed methodology in Data file 1—Table 1). The quality and quantity of the DNA were assessed by the Qubit fluorometer (Invitrogen, Carlsbad, CA, USA) and Infinite F200 microplate reader (TECAN), according to the manufacturer’s instruction. The status of the DNA was visually inspected by 0.8% agarose gel electrophoresis. Purified genomic DNA was sent for library preparation (detailed methodology in Data file 1—Table 1) and whole genome sequencing (WGS) at BGI Group (Shenzhen, Guangdong, China). A total of 40 Gb (Gigabase pair) (14-fold) of subread bases with a read length of 150 bp were generated using next-generation sequencing (NGS) technology on an Illumina HiSeq 2500 platform (detailed methodology in Data file 1—Table 1).

**Table 1:** Overview of data files/data sets

Label	Name of data file/data set	File types (file extension)	Data repository and identifier (DOI or accession number)
Data file 1	DNA isolation and library preparation methodology	.docx	https://figshare.com/s/67c8971f5a69277d55d0
Data file 2	Whole genome assembly data	FASTA	NCBI GeneBank (Accession numbers:GCA_004361675.1)(https://www.ncbi.nlm.nih.gov/assembly/GCA_004361675.1)
Data set 3	Whole genome sequence	FASTA	NCBI GeneBank (Accession numbers SMSF01000001- SMSF01003972) (https://www.ncbi.nlm.nih.gov/nuccore/SMSF00000000.1)
Data set 4	Annotation files	.tsv	https://figshare.com/s/a7873490048898ac1d56

After sequencing, quality of the raw sequencing reads were inspected using FastQC version 0.11.8 [4]. Reads were quality controlled including removing adaptor sequences, contamination and low-quality reads from raw reads using Trimmomatic V0.32 [5]. A total of 247,325,362 clean reads were included in the assembly. Subsequently, for de novo assembly we used ABySS v. 2.1.5 assembler [6], which generated 32,94,295 contigs (minimum contig size 200 bp). Next, ABACAS v.1.3.1 pipeline was used with the reference genome ARS1 (GCA_001704415.1) [7] to arranging, ordering, and orientation of the assembled genome [8]. The genome assembly data has been deposited in the NCBI GenBank under the Accession number GCA_001704415.1 (Data file 2—Table 1). The final assembled genome size of BBG is 3.04 Gb with 724.80 Mb (Megabase pair) gaps and GC content of 41.77%. Completeness of the genome was assessed with benchmarking universal single-copy orthologs (BUSCO) version 3.0.2 [9] which showed 82.5% completeness.

Genes were annotated using Maker version 3.0 pipeline [10] which identified 26,458 gene models. RepeatMasker V 4.0.9 [11] using the latest version of the repbase database [12] identified 31.85% repeat elements in the genome. Finally, InterProScan V 5.33–72.0 [13] was used to identify the gene ontology (GO) terms, which identified a total of 12,589 GO terms and 8173 genes have at least 1 associated GO term. The whole genome sequence data has been submitted in the NCBI GenBank under the Accession numbers SMSF01000001–SMSF01003972 (Data file 3—Table 1).

## Limitations

The number of unassembled regions in the genome is 3943 and the total number of bases placed in this gap is 724,808,570 bp.

## Data Availability

The genome sequence information has been accessible at DDBJ/ENA/GenBank under the Accession Numbers SMSF01000001–SMSF01003972 and the assembled genome at GCA_001704415.1. The version reported in this paper is the first version, SMSF00000000.1.

## Abbreviations

BBG: Black Bengal goat
GDP: gross domestic production
EDTA: ethylene diamine tetra-acetic acid
DNA: deoxyribonucleic acid
WGS: whole genome sequencing
BUSCO: benchmarking universal single-copy orthologs
ABACAS: algorithm-based automatic contiguation of assembled sequences
Gb: giga base pair
Mb: megabase pair
Kb: kilobase pair
bp: base pair
GO: gene ontology
gDNA: genomic DNA
PCR: polymerase chain reaction
